# Associations of total and type-specific physical activity with mortality in chronic obstructive pulmonary disease: a population-based cohort study

**DOI:** 10.1186/s12889-018-5167-5

**Published:** 2018-02-17

**Authors:** Sonia Wing Mei Cheng, Zoe McKeough, Jennifer Alison, Sarah Dennis, Mark Hamer, Emmanuel Stamatakis

**Affiliations:** 1 Discipline of Physiotherapy, The University of Sydney, Sydney, Australia; 2 Ingham Institute for Applied Medical Research, Sydney, Australia; 3 University of Loughborough, Loughborough, UK; 4 Charles Perkins Centre, School of Public Health, Prevention Research Collaboration, The University of Sydney, Sydney, Australia

**Keywords:** Chronic obstructive pulmonary disease, Physical activity, Mortality, Epidemiology

## Abstract

**Background:**

Regular physical activity is recommended for all people with chronic obstructive pulmonary disease (COPD), but the dose of physical activity required to gain mortality benefit in this population is not yet known. This aim of this study was to examine the associations of total and type-specific physical activity with mortality risk in people with COPD.

**Methods:**

People with COPD aged ≥40 years were identified from the 1997 Health Survey for England and the 1998 and 2003 Scottish Health Survey cohorts. Self-reported total physical activity, moderate-vigorous intensity physical activity (MVPA), walking, domestic physical activity, and sport/exercise were assessed at baseline. Cox proportional hazards models were used to examine the associations between physical activity and mortality risk.

**Results:**

Two thousand three hundred ninety-eight participants with COPD were included in the analysis and followed up for a mean 8.5 (SD 3.9) years. For both total physical activity and MVPA, we observed dose-response associations with all-cause and cardiovascular disease (CVD) mortality risk, and with respiratory mortality risk to a lesser extent. Compared to those who reported no physical activity, participants who met the physical activity guidelines demonstrated the greatest reductions in all-cause (HR 0.56, 95% CI 0.45–0.69), CVD (HR 0.48, 95% CI 0.32–0.71) and respiratory mortality risk (HR 0.40, 95% CI 0.24–0.67). Participants who reported a level of physical activity of at least half the dosage recommended by the guidelines also had a reduced risk of all-cause (HR 0.75, 95% CI 0.56–1.00) and CVD mortality (HR 0.48, 95% CI 0.26–0.88). Dose-response associations with mortality risk were demonstrated for walking and sport/exercise, but not domestic physical activity.

**Conclusions:**

We found a dose-response association between physical activity and all-cause and CVD mortality risk in people with COPD, with protective effects appearing at levels considerably lower than the general physical activity recommendations. People with COPD may benefit from engagement in low levels of physical activity, particularly walking and structured exercise.

**Electronic supplementary material:**

The online version of this article (10.1186/s12889-018-5167-5) contains supplementary material, which is available to authorized users.

## Background

Compared to their healthy peers, people with chronic obstructive pulmonary disease (COPD) engage in a lower intensity of daily physical activity (PA), and spend significantly more time sitting and lying down [1, 2]. This has been attributed to disease-specific limitations such as dyspnoea, fatigue and reduced exercise tolerance, which reduce the capacity of people with COPD to engage in moderate-vigorous intensity physical activity (MVPA) [3]. Previous cohort studies have consistently shown that low levels of PA are associated with an increased risk of all-cause and respiratory mortality in people with COPD, as well as an increased risk of COPD exacerbations, irrespective of the degree of airflow obstruction [4]. Low levels of PA are also associated with an increased risk of hospitalisation, re-hospitalisation after an acute exacerbation, and longer hospital length of stay [4, 5].

Although regular PA is recommended for all patients with COPD [6], there is a lack of high-quality evidence on which to base specific PA recommendations for this population, in particular the dose of PA required to gain mortality benefit. A Spanish cohort study of 611 people with COPD found that those who “took walks regularly for >8 km, no less than 5 days a week, or practised sports” had a lower mortality risk compared to those who “don’t leave the house and life is limited to the bed or armchair, or to doing some domestic chores” (risk ratio (RR) 0.38, 95% confidence interval (CI) 0.11–1.29) [7]. Another cohort study of 173 people with moderate to very severe COPD, which used accelerometry to measure PA levels, found that every ten vector-magnitude unit (VMU) increase in daily PA was associated with a 14% reduction in mortality risk [8]. Such studies are limited by the use of small non-representative samples of people with COPD to examine the association between PA and mortality risk, and by arbitrary definitions of what constitutes “low” and “high” levels of PA. Many different questionnaires and activity monitors have been used to assess PA in people with COPD, and the optimal mode of reporting (e.g., energy expenditure, number of steps/day) is not yet known [4, 9].

The considerable heterogeneity in the way that PA has been classified and reported in previous studies has made it difficult to develop PA guidelines specific to people with COPD, and subsequently clinicians must rely on the PA guidelines for the general population to inform clinical recommendations [10]. It is unknown whether specific types of PA, such as walking and domestic PA, are associated with reductions in mortality risk. Exploring types of PA that are relevant to functionally limited populations such COPD, and the dose of PA required to gain mortality benefit, is necessary in order to guide the prescription of PA in clinical practice and to inform interventions which aim to increase PA.

The aim of this study was to examine the associations between total and type-specific PA and mortality risk in a series of representative cohorts of adults with COPD, specifically:

1. The dose-response association between total PA and the risk of all-cause, cardiovascular disease (CVD), and respiratory mortality within the context of current public health recommendations; and
2. The dose-response association between specific types of PA and the risk of all-cause, CVD, and respiratory mortality.

## Methods

### Study design

The Health Survey for England (HSE) and Scottish Health Survey (SHS) cohorts are general population-based health examinations of individuals aged ≥16 years living in households in England and Scotland respectively. Participants were selected using a multistage stratified probability design, based on geographical location, to provide a nationally representative sample of the population in each constituent country. Further details on the HSE/SHS sampling methods can be found elsewhere [11, 12].

Participants for the present analysis were drawn from the HSE and SHS survey cohorts with available data on lung function and PA (HSE 1997 and SHS 1998, 2003), and were linked prospectively to National Health Service mortality data. The surveys consisted of two household visits: an interviewer visit, to collect information on physical and mental health and health behaviours, followed by a nurse visit to obtain physiological measures. Response rates for the interviewer visit ranged from 76 to 83%, and 31 to 44% for the subsequent nurse visit. Ethics approvals for the HSE and SHS were obtained from the London Research Ethics Council and Scotland Local Research Ethics Councils respectively. Participants gave written informed consent.

### Participants

There were two eligibility criteria to identify participants with COPD: (1) age ≥ 40 years, and (2) meeting the Global Initiative for Chronic Obstructive Lung Disease (GOLD) spirometric criteria for COPD (forced expiratory volume in one second (FEV_1_)/forced vital capacity (FVC) ratio of < 0.7) [3]. Participants were followed-up to the time of death or, if there was no record of an event, data were censored at February 15, 2011 (HSE) and at December 31, 2009 (SHS).

### Measurements

#### Participant characteristics

Interviewers used computer-assisted interviewing modules to collect information on participants’ demographic and socioeconomic characteristics, self-reported health status, and other health behaviours (e.g., smoking, alcohol consumption).

#### Lung function

Spirometry was performed using a Vitalograph® Escort spirometer, which was calibrated prior to each participant’s use. Participants who normally required bronchodilator medication prior to strenuous exercise were allowed to take their usual medication before spirometry was performed. No additional bronchodilators were given by the survey nurses. Participants performed at least one practice attempt, followed by five attempts or until they were deemed too tired to continue. The best of the five attempts (excluding any technically unsatisfactory attempts, e.g., coughing during the blow, air leak around the mouthpiece) was used as the final result. The full spirometry protocol has been published elsewhere [13].

#### Physical activity

The Physical Activity and Sedentary Behaviour Assessment Questionnaire (PASBAQ) was used to collect data on physical activity. Participants reported the frequency (number of days in the last 4 weeks) and duration (of an average episode) of participation in four domains of PA: (1) “light” (e.g. general tidying) and “heavy” (e.g. spring cleaning) domestic activity; (2) “light” and “heavy” manual work, gardening and “do-it-yourself” activities; (3) light-intensity (slow/average pace) and moderate-intensity (fairly brisk/fast pace) walking; and (4) sport/exercise. Intensity of sport/exercise was determined by whether the activity had made participants “out of breath or sweaty”, and by the nature of the activity as indexed in the metabolic equivalent (MET) compendium [14]. The PASBAQ has demonstrated satisfactory validity when compared against accelerometry data in a representative sample of British adults; Spearman’s correlation coefficients were 0.20 (95% confidence interval (CI) 0.25–0.35) in men and 0.30 (95% CI 0.15–0.26) in women [15]. Participation in PA was then calculated in MET-hours/week by multiplying the volume of activity (frequency x duration) by the intensity of the activity in METs [14].

Five PA variables were derived for the analyses: MET-hours/week of total non-occupational PA, walking, domestic PA, and sport/exercise, and minutes/week of MVPA. Total PA and MVPA were converted into four categories based on adherence to the general PA recommendations (i.e., at least 150 min/week of moderate-intensity aerobic PA (MPA), or at least 75 min/week of vigorous-intensity aerobic PA (VPA), or an equivalent combination of MVPA) [10]. This recommendation is equivalent to 7.5 MET-hours/week of PA [16]. Participants were classified as ‘Inactive’ (0 MET-hours/week of total PA, 0 min/week of MVPA), ‘Insufficiently Active (Low)’ (< 3.75 MET-hours/week of total PA, < 75 min/week of MPA or equivalent combination of MVPA), ‘Insufficiently Active (High)’ (3.75 to < 7.5 MET-hours/week of total PA, 75 to < 150 min/week of MPA or equivalent combination of MVPA), or ‘Sufficiently Active’ (≥7.5 MET-hours/week of PA, ≥150 min/week of MPA or equivalent combination of MVPA). Participants who reported no PA were specified as the referent group. This method of classifying PA has been used in other cohort studies of people with COPD [5].

The walking, domestic PA, and sport/exercise variables were converted into three categories. The referent group was specified as no participation in walking, domestic PA, or sport/exercise respectively (‘No PA’). For each type of PA, the remaining participants were divided into one of two groups: below the median level of activity of the cohort (‘Low PA’), or equal to or above the median level of activity of the cohort (‘High PA’). The medians were 5.25 MET-hours/week for walking, 5.70 MET-hours/week for domestic PA, and 8.00 MET-hours/week for sport/exercise. Previous studies have used this approach to distribute participants evenly into each category and ensure that the Cox proportional hazards assumption is met [17].

#### Mortality

The study outcomes were all-cause mortality, CVD mortality, and respiratory mortality. The primary cause of death was identified from the death certificate and from any additional information provided by the certifying physician, and was recorded according to the International Classification of Diseases (ICD) Ninth and Tenth Revisions. CVD codes were 390 to 459 (ICD-9) and I01 to I99 (ICD-10), and respiratory codes 460 to 519 (ICD-9) and J00 to J99 (ICD-10).

### Covariates

Potential confounders were identified a priori from a review of current literature [5, 16, 18] and the most parsimonious selection of variables that achieved the greatest control of confounding was used. The following covariates were included in the analyses: age, sex, ethnicity (Caucasian, non-Caucasian), severity of COPD (mild, moderate, severe, or very severe according to the GOLD stages [3]), history of CVD at baseline, history of cancer at baseline, history of diabetes at baseline, self-reported longstanding illness, body mass index (BMI), smoking status (current smoker, ex-smoker, never smoked), age finished full-time education (< 14 years, 15–18 years, ≥19 years), and alcohol consumption (does not drink, less than once a week, 1–4 times/week, ≥5 times/week).

### Statistical analyses

Differences in the distribution of baseline characteristics were examined using chi-square tests for categorical variables and analysis of variance (ANOVA) for continuous variables. Cox proportional hazards regression models were used to compute hazard ratios (HR) with 95% CIs and compare mortality risk across the categories of PA. The proportional hazards assumption was examined by comparing the cumulative hazard plots of the five PA variables. No appreciable violations were noted. Covariates were added to the model in three stages: age, sex and ethnicity (Model 1); plus severity of COPD, history of CVD, history of cancer, history of diabetes, self-reported longstanding illness, and BMI (Model 2); plus smoking status, age finished full time education, and alcohol consumption (Model 3). Analyses of walking, domestic PA and sport/exercise were also mutually adjusted for the other types of PA. Participants with missing data for mortality (*n* = 243) or for any of the covariates (*n* = 490) were excluded from the analyses.

Two sensitivity analyses were performed. To minimise the potential for reverse causality in the main analysis (i.e., that participants had an increased risk of mortality because of pre-existing medical conditions rather than their PA levels), the first excluded those who had died within the first 12 months of follow-up (*n* = 49), and those with existing CVD (*n* = 339), cancer (*n* = 135), and diabetes (*n* = 123) at baseline. Additional sensitivity analyses were conducted to exclude those who had died within the first 2 years of follow-up (*n* = 124) and within the first 3 years of follow-up (*n* = 196). As no appreciable differences were found, these participants were retained in the main analyses. To assess for misclassification of a diagnosis of COPD, the second excluded participants who had never smoked (*n* = 664), those with a history of asthma (*n* = 243), and those who reported a respiratory infection in the 3 weeks prior to the first household visit (*n* = 353). All statistical tests were two-sided, with a *p*-value < 0.05 considered statistically significant. All analyses were performed using SPSS Version 22.0 (IBM Corp, USA).

## Results

Of the 19,000 participants with available data on lung function and PA, 2398 participants met the eligibility criteria for COPD and were included in the analysis. The mean age of the sample was 62.6 (SD 11.5) years and 52% were men. There was a high prevalence of COPD of mild (42.7%) and moderate (37.5%) severity, and of never-smokers (27.9%). Baseline characteristics, grouped according to the degree of adherence to the general PA recommendations, are displayed in Table 1. Participants who reported no PA were older, had COPD of greater severity, had more medical comorbidities, and had a lower level of education (*p* < 0.001). Mortality data were available for 2155 participants. Over a mean 8.5 (3.9) years follow-up, there were 571 deaths (26%) of which 172 were CVD-related deaths and 105 were respiratory-related deaths, corresponding to a total of 18,222 person years.

**Table 1:** Baseline characteristics of 2398 participants with COPD recruited from the Healthy Survey of England (1997) and Scottish Health Survey (1998, 2003)

Variable	All participants (*n* = 2398)	Inactive^d^ (*n* = 489)	Insufficiently Active (Low)^e^ (*n* = 386)	Insufficiently Active (High)^f^ (*n* = 225)	Sufficiently Active^g^ (*n* = 1268)	*p*-value
Mean age, years	62.6 (11.5)	67.9 (11.6)	63.8 (10.9)	62.5 (11.3)	60.1 (11.0)	< 0.001
Men, %	52.1	54.6	47.9	47.1	53.5	0.06
Caucasian, %	98.9	98.8	98.7	99.2	99.0	0.92
FEV_1_% predicted^a^, %						< 0.001
GOLD I: ≥80% pred.	42.7	26.0	37.8	41.1	49.7	
GOLD II: 50% ≥ FEV_1_% < 80% pred.	37.5	40.5	41.2	39.0	35.2	
GOLD III: 30% ≥ FEV_1_% < 50% pred.	14.5	25.2	15.4	15.8	10.6	
GOLD IV: FEV_1_ < 30% pred.	5.3	8.3	5.6	4.1	4.5	
History of CVD^b^, %	14.1	28.4	18.7	11.8	7.7	< 0.001
History of cancer, %	5.6	6.1	6.0	5.1	5.4	0.91
History of diabetes^c^, %	5.1	10.0	7.3	4.7	2.7	< 0.001
Self-reported longstanding illness, %	62.2	85.5	69.2	61.2	51.3	< 0.001
Self-reported asthma, %	10.1	13.9	10.6	9.8	8.6	0.01
Mean BMI, kg/m^2^	26.6 (4.7)	27.3 (5.5)	27.1 (4.9)	26.6 (5.0)	26.3 (4.3)	0.001
Smoking status, %						< 0.001
Current	37.2	42.7	38.6	38.6	34.4	
Ex	34.9	37.1	33.2	36.2	34.3	
Never	27.9	20.2	28.2	25.2	31.3	
Aged finished full-time education, %						< 0.001
≤ 14 years	28.7	45.7	32.6	27.1	21.2	
15–18 years	59.4	48.2	57.8	61.2	63.8	
19 years and older	12.0	6.1	9.6	11.8	15.0	
Alcohol consumption, %						< 0.001
Does not drink	13.1	22.5	13.5	11.4	9.6	
Less than once per week	26.9	27.4	31.9	30.2	24.6	
1–4 times/week	37.4	29.2	35.0	35.7	41.6	
≥ 5 times/week	22.6	20.9	19.7	22.7	24.1	

Values are means (SD) unless otherwise stated

*FEV*_*1*_ forced expiratory volume in 1 second, *CVD* cardiovascular disease, *BMI* body mass index

^a^COPD severity was classified according to Global Initiative for Lung Disease (GOLD) stages

^b^A history of cardiovascular disease was defined as self-reported or doctor-diagnosed angina, heart attack or stroke

^c^A history of diabetes was defined as self-reported or doctor-diagnosed diabetes, and glycated haemoglobin (HBa1C) ≥6.5%

^d^Participants who reported no physical activity during the week

^e^Participants who reported < 3.75 MET-hours/week of physical activity

^f^Participants who reported between 3.75 and < 7.5 MET-hours/week of physical activity

^g^Participants who reported ≥7.5 MET-hours/week of physical activity as per current public health recommendations

**Fig. 1:**
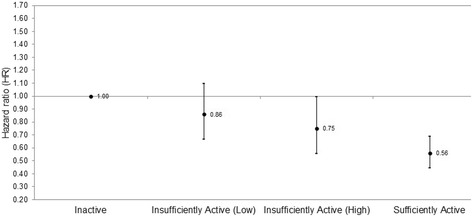
The association of total physical activity with all-cause mortality risk in participants with COPD (*n* = 2155). The figure shows the dose-response association of total physical activity (PA) with all-cause mortality risk based on adherence to the general PA recommendations. Participants were classified as ‘Inactive’ (0 MET-hours/week of total PA), ‘Insufficiently Active (Low)’ (< 3.75 MET-hours/week of total PA), ‘Insufficiently Active (High)’ (3.75 to < 7.5 MET-hours/week of total PA), or ‘Sufficiently Active’ (≥7.5 MET-hours/week of PA)

**Fig. 2:**
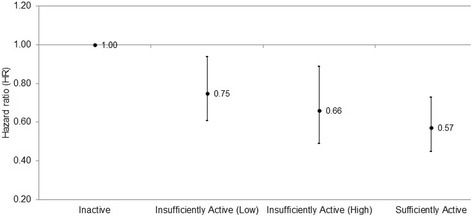
The association of moderate-vigorous intensity physical activity with all-cause mortality risk in participants with COPD (*n* = 2155). The figure shows the dose-response association of moderate-vigorous physical activity (MVPA) with all-cause mortality risk based on adherence to the general physical activity (PA) recommendations. Participants were classified as ‘Inactive’ (0 min/week of MVPA), ‘Insufficiently Active (Low)’ (< 75 min/week of moderate-intensity PA or equivalent combination of MVPA), ‘Insufficiently Active (High)’ (75 to < 150 min/week of moderate-intensity PA or equivalent combination of MVPA), or ‘Sufficiently Active’ (≥150 min/week of moderate-intensity PA or equivalent combination of MVPA)

The associations of MET-hours/week of total PA and minutes/week of MVPA with mortality risk are shown in Table 2. Dose-response associations with all-cause, CVD and respiratory mortality risk were demonstrated for total PA and MVPA. Participants in the ‘Insufficiently Active (High)’ group for total PA had a reduced risk of all-cause and CVD mortality compared to those who reported no PA, but no significant mortality risk reductions were observed in the ‘Insufficiently Active (Low)’ group for total PA (Fig. 1). Conversely, participants in the ‘Insufficiently Active (Low)’ group for MVPA had a reduced risk of all-cause mortality compared to those who reported no MVPA (Fig. 2). These dose-response associations were also observed in the sensitivity analyses (Additional file 1: Tables S1 and S2).

**Table 2:** The associations of total physical activity and moderate-vigorous intensity physical activity with mortality risk in participants with COPD (*n* = 2155)

	Total PA				MVPA			
	Cases/No.	Model 1 HR (95% CI)	Model 2 HR (95% CI)	Model 3 HR (95% CI)	Cases/No.	Model 1 HR (95% CI)	Model 2 HR (95% CI)	Model 3 HR (95% CI)
All-cause mortality (571 deaths)								
Inactive^a^	171/452	1.00	1.00	1.00	289/815	1.00	1.00	1.00
Insufficiently Active (Low)^b^	114/355	0.75 (0.59–0.95)	0.81 (0.64–1.03)	0.86 (0.67–1.10)	122/418	0.68 (0.55–0.84)	0.73 (0.59–0.90)	0.75 (0.61–0.94)
Insufficiently Active (High)^c^	68/222	0.60 (0.46–0.80)	0.70 (0.53–0.94)	0.75 (0.56–1.00)	53/229	0.55 (0.42–0.74)	0.63 (0.47–0.85)	0.66 (0.49–0.89)
Sufficiently Active^d^	218/1126	0.43 (0.35–0.52)	0.51 (0.41–0.63)	0.56 (0.45–0.69)	107/693	0.45 (0.36–0.57)	0.52 (0.41–0.66)	0.57 (0.45–0.73)
*P* trend		< 0.001	< 0.001	< 0.001		< 0.001	< 0.001	< 0.001
CVD mortality (172 deaths)								
Inactive	61/452	1.00	1.00	1.00	97/815	1.00	1.00	1.00
Insufficiently Active (Low)	38/355	0.72 (0.48–1.07)	0.78 (0.52–1.18)	0.80 (0.53–1.21)	35/418	0.59 (0.40–0.86)	0.68 (0.46–1.01)	0.69 (0.47–1.03)
Insufficiently Active (High)	14/222	0.36 (0.20–0.64)	0.47 (0.26–0.86)	0.48 (0.26–0.88)	15/229	0.48 (0.28–0.84)	0.62 (0.36–1.08)	0.65 (0.37–1.13)
Sufficiently active	59/1126	0.34 (0.23–0.48)	0.45 (0.31–0.67)	0.48 (0.32–0.71)	25/693	0.33 (0.21–0.51)	0.42 (0.27–0.68)	0.46 (0.29–0.73)
*P* trend		< 0.001	< 0.001	< 0.001		< 0.001	0.002	0.005
Respiratory mortality (105 deaths)								
Inactive	37/452	1.00	1.00	1.00	56/815	1.00	1.00	1.00
Insufficiently Active (Low)	19/355	0.57 (0.33–0.99)	0.71 (0.40–1.26)	0.79 (0.45–1.41)	27/418	0.73 (0.46–1.15)	0.76 (0.47–1.22)	0.86 (0.53–1.39)
Insufficiently Active (High)	19/222	0.71 (0.41–1.25)	0.86 (0.49–1.52)	0.97 (0.52–1.68)	6/229	0.30 (0.13–0.70)	0.39 (0.16–0.91)	0.43 (0.18–1.02)
Sufficiently Active	30/1126	0.26 (0.16–0.42)	0.34 (0.20–0.56)	0.40 (0.24–0.67)	16/693	0.33 (0.19–0.58)	0.39 (0.22–0.69)	0.45 (0.25–0.81)
*P* trend		< 0.001	< 0.001	0.001		< 0.001	0.002	0.007

*PA* physical activity, *MVPA* moderate-vigorous intensity physical activity, *HR* hazard ratio, *CVD* cardiovascular disease

Model 1: adjusted for age and sex; Model 2: also adjusted for COPD severity, history of cardiovascular disease, history of cancer, history of diabetes, self-reported longstanding illness and body mass index; Model 3: also adjusted for smoking status, education level and alcohol consumption

^a^Participants who reported no physical activity during the week; participants who reported no moderate-vigorous intensity physical activity during the week

^b^Participants who reported < 3.75 MET-hours/week of physical activity; participants who reported < 75 min/week of moderate-intensity physical activity, or < 32.5 min/week of vigorous-intensity physical activity, or an equivalent combination of moderate-vigorous intensity physical activity

^c^Participants who reported between 3.75 and < 7.5 MET-hours/week of physical activity; participants who reported between 75 and < 150 min/week of moderate-intensity physical activity, or between 32.5 and < 75 min/week of vigorous-intensity physical activity, or an equivalent combination of moderate-vigorous intensity physical activity

^d^Participants who adhered to the current recommendation of ≥7.5 MET-hours/week of physical activity; participants who adhered to the current recommendation of ≥150 min/week of moderate-intensity physical activity, or ≥75 min/week of vigorous-intensity physical activity, or an equivalent combination of moderate-vigorous intensity physical activity

The associations of MET-hours/week of walking, domestic PA, and sport/exercise with mortality risk are shown in Table 3. Dose-response associations with all-cause, CVD and respiratory mortality risk were demonstrated for walking, with no significant mortality risk reductions observed in the ‘low’ walking group. There was a dose-response association between sport/exercise and all-cause mortality risk, but not CVD or respiratory mortality risk. The few number of CVD and respiratory deaths in the ‘low’ and ‘high’ categories of sport/exercise have likely contributed to this result. Domestic PA of any level was not associated with reductions in mortality risk. Similar results were demonstrated in the sensitivity analyses (Additional file 1: Tables S3 and S4).

**Table 3:** The associations of type-specific physical activity with mortality risk in participants with COPD (*n* = 2155)

	Walking				Domestic PA				Sport/exercise			
	Cases/No.	Model 1 HR (95% CI)	Model 2 HR (95% CI)	Model 3 HR (95% CI)	Cases/No.	Model 1 HR (95% CI)	Model 2 HR (95% CI)	Model 3 HR (95% CI)	Cases/No.	Model 1 HR (95% CI)	Model 2 HR (95% CI)	Model 3 HR (95% CI)
All-cause mortality (571 deaths)												
No PA^a^	245/733	1.00	1.00	1.00	320/1040	1.00	1.00	1.00	473/1556	1.00	1.00	1.00
Low PA^b^	186/708	0.71 (0.58–0.85)	0.79 (0.65–0.96)	0.88 (0.72–1.08)	133/529	0.70 (0.57–0.86)	0.77 (0.62–0.94)	0.86 (0.70–1.06)	49/288	0.64 (0.48–0.87)	0.68 (0.51–0.92)	0.77 (0.57–1.05)
High PA^c^	140/714	0.52 (0.42–0.64)	0.61 (0.49–0.76)	0.70 (0.56–0.88)	118/586	0.67 (0.54–0.82)	0.75 (0.60–0.93)	0.85 (0.68–1.07)	49/311	0.53 (0.39–0.71)	0.59 (0.44–0.80)	0.69 (0.51–0.93)
*P* trend		< 0.001	< 0.001	0.002		0.001	0.027	0.234		< 0.001	0.004	0.037
CVD mortality (172 deaths)												
No PA	86/733	1.00	1.00	1.00	106/1040	1.00	1.00	1.00	152/1556	1.00	1.00	1.00
Low PA	51/708	0.56 (0.39–0.79)	0.66 (0.47–0.94)	0.72 (0.50–1.03)	31/529	0.50 (0.34–0.75)	0.61 (0.40–0.92)	0.70 (0.46–1.06)	10/288	0.42 (0.22–0.80)	0.43 (0.23–0.82)	0.50 (0.26–0.96)
High PA	35/714	0.38 (0.25–0.56)	0.49 (0.33–0.74)	0.56 (0.36–0.85)	35/586	0.62 (0.42–0.91)	0.78 (0.52–1.16)	0.94 (0.62–1.42)	10/311	0.34 (0.18–0.64)	0.43 (0.23–0.83)	0.50 (0.26–0.96)
*P* trend		< 0.001	0.002	0.012		0.071	0.431	0.972		0.006	0.046	0.096
Respiratory mortality (105 deaths)												
No PA	50/733	1.00	1.00	1.00	60/1040	1.00	1.00	1.00	93/1556	1.00	1.00	1.00
Low PA	37/708	0.67 (0.44–1.03)	0.77 (0.50–1.18)	0.92 (0.58–1.47)	30/529	0.79 (0.51–1.23)	0.80 (0.51–1.26)	1.13 (0.70–1.84)	4/288	0.27 (0.10–0.74)	0.34 (0.12–0.92)	0.44 (0.16–1.20)
High PA	18/714	0.32 (0.18–0.54)	0.38 (0.22–0.67)	0.47 (0.26–0.85)	15/586	0.43 (0.25–0.76)	0.49 (0.27–0.87)	0.63 (0.34–1.15)	8/311	0.44 (0.21–0.90)	0.49 (0.24–1.03)	0.73 (0.34–1.54)
*P* trend		< 0.001	0.001	0.009		0.005	0.018	0.098		0.134	0.203	0.690

*PA* physical activity, *HR* hazard ratio, *CVD* cardiovascular disease

Model 1: adjusted for age and sex; Model 2: also adjusted for COPD severity, history of cardiovascular disease, history of cancer, history of diabetes, self-reported longstanding illness and body mass index; Model 3: also adjusted for smoking status, education level, alcohol consumption and the two other type-specific PA variables

^a^“No PA” was defined as no self-reported walking during the week, no self-reported domestic physical activity during the week, and no self-reported sport/exercise during the week

^b^“Low PA” was defined as < 5.25 MET-hours/week of walking, < 5.70 MET-hours/week of domestic physical activity, and < 8.00 MET-hours/week of sport/exercise

^c^“High PA” was defined as ≥5.25 MET-hours/week of walking, ≥5.70 MET-hours/week of domestic physical activity, and ≥8.00 MET-hours/week of sport/exercise

## Discussion

This study of 2398 participants who met the GOLD spirometric criteria for COPD provides a detailed analysis of the dose-response associations between PA and mortality risk within the context of current public health recommendations, and for specific types of PA. The use of established thresholds to examine PA in both MET-hours/week and minutes/week provides clinicians with a meaningful reference standard to guide prescription of PA, and enables future studies to make direct comparisons to the magnitude of mortality risk described in this sample. This is the first population-based cohort study of people with COPD to examine whether specific types of PA confer mortality benefit, which has high clinical relevance when developing interventions to increase PA in people with COPD.

Dose-response associations with mortality risk were demonstrated for total PA, MVPA, walking and sport/exercise, but not domestic PA. A previous Danish population-based cohort study of people with COPD, which compared subjects who reported low, moderate, or high levels of PA to subjects who reported a very low level of PA, showed that some level of regular PA was associated with a lower risk of all-cause and respiratory mortality [18]. In our study, reductions in mortality risk of the greatest magnitude were observed in participants who adhered to the PA guidelines. Compared to those who reported no PA, participants who were ‘Sufficiently Active’ showed a 45% reduction in all-cause mortality risk, a 50–55% reduction in CVD mortality risk, and a 55–60% reduction in respiratory mortality risk.

Although there is added benefit in adhering to the PA guidelines, meeting such recommendations is often difficult for people with symptomatic COPD. This study showed that participants who did not meet the guidelines but reported at least 3.75 MET-hours/week of total PA, or reported some level of MVPA, still had significant reductions in mortality risk. After taking the sensitivity analyses into consideration, which yielded similar mortality risk reductions in participants without existing CVD, cancer and diabetes, these results suggest that the dose of PA required to gain mortality benefit in COPD may be lower than is currently estimated. This is consistent with previous observations that relatively low levels of PA have beneficial effects on mortality risk in people with COPD. It has been suggested that as little as 2 h/week of walking or cycling is associated with a 30–40% risk reduction in respiratory mortality and COPD hospitalisation [18].

In both the main and sensitivity analyses, participants in the ‘Insufficiently Active (Low)’ group for MVPA had a significant reduction in all-cause mortality risk (HR 0.75, 95% CI 0.61–0.94). However, no significant reductions in all-cause mortality risk were observed in participants who reported < 3.75 MET-hours/week of total PA (HR 0.86, 95% CI 0.67–1.10). This subtle difference between the two equivalent measures of PA is likely due to error in the calculation of MET-hours/week of total PA, in which the intensity of PA is approximated according to the nature of the activity as indexed in the MET compendium. Whether higher intensity PA has beneficial effects on mortality risk independent of the volume of activity is not yet known in people with COPD. A Spanish cohort study demonstrated a 20% reduction in the risk of COPD hospitalisation for every additional 1000 daily steps at low average intensity, but no change in hospitalisation risk with additional steps at high average intensity [19]. Conversely, higher intensity PA was associated with a lower prevalence of metabolic syndrome in 223 subjects with COPD drawn from the National Health and Nutrition Examination Survey 2003–2006 dataset [20]. Considering the innate challenges of increasing MVPA in people with COPD, encouraging more light-intensity PA in this population may be a first and more realistic step than targeting MVPA alone.

Although there was a dose-response association between sport/exercise and mortality risk, sport is not feasible for most people with COPD due to the older age of the population and the high prevalence of comorbidities such as poly-pharmacy and mobility problems [21], which are associated with poorer physical performance [22]. Walking, however, is an achievable and accessible type of PA. It requires no specialised exercise equipment and, in the form of ground walking or treadmill walking, is commonly undertaken as part of pulmonary rehabilitation programs with good effect [23]. This study showed that participants who reported at least 5.25 MET-hours/week of walking (approximately equivalent to 105 min/week of brisk walking) had a 30% reduction in all-cause mortality risk, a 44% reduction in CVD mortality risk, and a 53% reduction in respiratory mortality risk compared to those who reported no walking. Future studies should explore strategies for incorporating more walking into daily life for people with COPD. Nordic walking is one such strategy, where improvements in PA were maintained to 3 months in the group of participants [24].

Although domestic PA is feasible for most people with COPD, this study showed no association between domestic PA of any level and mortality risk. There is conflicting evidence about the protective effect of domestic PA on mortality. The domain is often poorly defined; for example, housework and gardening are both treated as domestic PA despite most housework activities being classified as light intensity, since they do not require locomotion or the use of large muscles groups [25]. An English population-based study of 15,000 adults found that PA “around the home”, such as lawn mowing and digging, was inversely related to all-cause mortality risk [26]. However, a recent study of the SHS cohort found no association between intense domestic PA and CVD mortality risk after adjusting for relevant confounders [17]. The inclusion of more light intensity activities in our definition of domestic PA may explain the absence of a dose-response association between domestic PA and mortality risk.

Our study has several limitations. A fixed FEV_1_/FVC ratio was used to identify participants with COPD due to a lack of other information that may have helped to confirm a diagnosis. Although COPD can be identified with high diagnostic accuracy using spirometry, a fixed ratio of obstruction would not take into account any reversible factors that may have influenced FEV_1_, such as asthma or a respiratory infection on the day of testing. Spirometry performed on 219 adults in a general practice setting showed a sensitivity of 0.92 (95% CI 0.80–0.97) for diagnosing COPD, with eight “false positives” identified from further laboratory testing [27]. To account for misclassification of COPD in our study, we conducted sensitivity analyses which excluded participants with asthma and/or a recent respiratory infection, and these yielded similar results to the main analysis.

Misclassification of the PA variables is also possible, since self-reported methods were used to quantify PA and any changes in PA levels during follow-up were not accounted for. This would have most likely reduced the magnitude of the effect of PA on mortality risk [28]. It is well-established that self-report PA questionnaires tend to overestimate the time spent physically active [29] as they are reliant on respondent recall and are often subject to social desirability bias [30], and thus should be interpreted with caution. Nevertheless, self-report questionnaires are frequently used to estimate PA levels in national populations for reasons of feasibility, cost-effectiveness, and to obtain information on specific types and domains of PA, which are not captured by objective measures. In comparison with accelerometry, the PASBAQ has demonstrated criterion validity similar to that of other questionnaires used in national surveys [15]. While there is no universally acceptable level for the magnitude of criterion validity coefficients for self-report PA questionnaires versus accelerometry, multiple reviews have indicated that correlations rarely exceed 0.40 [31–33].

Smoking history was not used as an eligibility criterion to identify participants with COPD due to a lack of available data to quantify smoking history in pack-years, and to preserve sample size. While the high prevalence of never-smokers in our sample (27.9%) is surprising, it is supported by a recent case-finding study of 74,818 patients with COPD showing that 20–26% of patients were never-smokers [34]. Exposure to second-hand smoke, pollutants, and occupational exposure to dusts and chemicals are other risk factors for COPD [3], and may explain the high proportion of never-smokers in the sample. Furthermore, sensitivity analyses excluding never-smokers yielded similar results to the main analysis. It is therefore unlikely that the inclusion of never-smokers in the analysis would have biased the magnitude of the effect of PA on mortality risk.

Finally, given that the HSE and SHS are general population-based health examinations and are not specifically designed to investigate people with COPD, some potential confounders may not have been captured and this may limit the generalisability of the results. These include history of previous exacerbations, COPD hospitalisations, exercise capacity at baseline, and severity of symptoms, which are known predictors of mortality [35–37]. The high proportion of mild and moderate COPD in the sample may also limit the generalisability of the results to people with COPD of greater severity.

## Conclusions

We observed a linear dose-response association between PA and all-cause, CVD and respiratory mortality risk in people with COPD. There is added mortality benefit in adhering to the PA guidelines, although engaging in PA above a threshold of 3.75 MET-hours/week (at least 75 min/week of MPA, 32.5 min of VPA, or an equivalent combination of MVPA) is still associated with significant reductions in mortality risk. Walking and structured exercise should be encouraged in people with COPD, but the effects of domestic PA on mortality remain unclear. Our results reinforce the importance of promoting regular PA as part of routine management of COPD, and the need for effective strategies to increase PA in this population.

## Additional file

Additional file 1: **Tables S1–S4.** The results of the sensitivity analyses are shown in the following supplementary tables. Table S1 shows the associations of total physical activity and moderate-vigorous intensity physical activity with mortality risk in participants with COPD without existing cardiovascular disease, cancer and diabetes at baseline. **Table S2.** shows the associations of total physical activity and moderate-vigorous intensity physical activity with mortality risk in participants with COPD with a smoking history but no coexisting asthma or recent respiratory infection. **Table S3.** shows the associations of type-specific physical activity with mortality risk in participants with COPD without existing cardiovascular disease, cancer and diabetes at baseline. **Table S4.** shows the associations of type-specific physical activity with mortality risk in participants with COPD with a smoking history but no coexisting asthma or recent respiratory infection. (DOCX 47 kb)

## Abbreviations

ANOVA: Analysis of variance
BMI: Body mass index
CI: Confidence interval
COPD: Chronic obstructive pulmonary disease
CVD: Cardiovascular disease
FEV_1_: Forced expiratory volume in one second
FVC: Forced vital capacity
GOLD: Global Initiative for Chronic Obstructive Lung Disease
HR: Hazard ratio
HSE: Health Survey for England
ICD: International Classification of Diseases
MET: Metabolic equivalent
MPA: Moderate-intensity physical activity
MVPA: Moderate-vigorous intensity physical activity
PA: Physical activity
PASBAQ: Physical Activity and Sedentary Behaviour Assessment Questionnaire
RR: Risk ratio
SHS: Scottish Health Survey
VMU: Vector-magnitude unit
VPA: Vigorous-intensity physical activity
