# Supplementary material for: Associations of total and type-specific physical activity with mortality in chronic obstructive pulmonary disease: a population-based cohort study
Source: BMC Public Health. 2018 Feb 17;18:268. doi: 10.1186/s12889-018-5167-5 (PMC5816365; doi:10.1186/s12889-018-5167-5)
Supplement: Supplementary file 1 — Tables S1–S4. The results of the sensitivity analyses are shown in the following supplementary tables. Table S1 shows the associations of total physical activity and moderate-vigorous intensity physical activity with mortality risk in participants with COPD without existing cardiovascular disease, cancer and diabetes at baseline. Table S2. shows the associations of total physical activity and moderate-vigorous intensity physical activity with mortality risk in participants with COPD with a smoking history but no coexisting asthma or recent respiratory infection. Table S3. shows the associations of type-specific physical activity with mortality risk in participants with COPD without existing cardiovascular disease, cancer and diabetes at baseline. Table S4. shows the associations of type-specific physical activity with mortality risk in participants with COPD with a smoking history but no coexisting asthma or recent respiratory infection. (DOCX 47 kb) [file 12889_2018_5167_MOESM1_ESM.docx]

**Additional file 1**

**Table S1.** Sensitivity analysis of the associations of total physical activity and moderate-vigorous intensity physical activity with mortality risk in participants with COPD without existing cardiovascular disease, cancer and diabetes at baseline^1^ (n=1635)

|  | Total PA | | | | MVPA | | | |
| --- | --- | --- | --- | --- | --- | --- | --- | --- |
|  | **Cases/No.** | **Model 1**  **HR (95% CI)** | **Model 2**  **HR (95% CI)** | **Model 3**  **HR (95% CI)** | **Cases/No.** | **Model 1**  **HR (95% CI)** | **Model 2**  **HR (95% CI)** | **Model 3**  **HR (95% CI)** |
| All-cause mortality  (391 deaths) | | |  |  |  |  |  |  |
| Inactive ^2^ | 99/265 | 1.00 | 1.00 | 1.00 | 175/516 | 1.00 | 1.00 | 1.00 |
| Insufficiently Active (Low) ^3^ | 79/249 | 0.82 (0.61-1.10) | 0.96 (0.71-1.30) | 1.04 (0.77-1.41) | 90/320 | 0.69 (0.53-0.88) | 0.73 (0.57-0.95) | 0.77 (0.59-0.99) |
| Insufficiently Active (High)^4^ | 49/173 | 0.54 (0.38-0.76) | 0.62 (0.44-0.88) | 0.67 (0.47-0.96) | 36/186 | 0.49 (0.34-0.70) | 0.53 (0.37-0.77) | 0.56 (0.39-0.81) |
| Sufficiently Active^4^ | 164/948 | 0.43 (0.34-0.56) | 0.51 (0.40-0.67) | 0.57 (0.44-0.74) | 90/613 | 0.50 (0.38-0.64) | 0.55 (0.42-0.71) | 0.60 (0.46-0.78) |
| *P* trend |  | <0.001 | <0.001 | <0.001 |  | <0.001 | <0.001 | 0.001 |
| CVD mortality  (101 deaths) | |  |  |  |  |  |  |  |
| Inactive | 28/265 | 1.00 | 1.00 | 1.00 | 49/516 | 1.00 | 1.00 | 1.00 |
| Insufficiently Active (Low) | 24/249 | 0.91 (0.53-1.57) | 0.96 (0.55-1.68) | 1.02 (0.58-1.78) | 23/320 | 0.63 (0.38-1.03) | 0.63 (0.38-1.04) | 0.65 (0.39-1.07) |
| Insufficiently Active (High) | 8/173 | 0.32 (0.15-0.71) | 0.34 (0.15-0.74) | 0.34 (0.15-0.77) | 8/186 | 0.41 (0.19-0.86) | 0.41 (0.19-0.86) | 0.41 (0.19-0.88) |
| Sufficiently Active | 41/948 | 0.41 (0.25-0.66) | 0.43 (0.26-0.71) | 0.45 (0.27-0.76) | 21/613 | 0.43 (0.26-0.72) | 0.44 (0.26-0.75) | 0.48 (0.29-0.82) |
| *P* trend |  | <0.001 | <0.001 | <0.001 |  | 0.006 | 0.010 | 0.022 |
| Respiratory mortality  (87 deaths) | |  |  |  |  |  |  |  |
| Inactive | 28/265 | 1.00 | 1.00 | 1.00 | 44/516 | 1.00 | 1.00 | 1.00 |
| Insufficiently Active (Low) | 15/249 | 0.54 (0.29-1.02) | 0.80 (0.42-1.52) | 0.89 (0.46-1.71) | 22/320 | 0.63 (0.38-1.05) | 0.69 (0.41-1.18) | 0.75 (0.44-1.29) |
| Insufficiently Active (High) | 18/173 | 0.65 (0.36-1.18) | 0.97 (0.52-1.82) | 1.06 (0.56-2.00) | 6/186 | 0.31 (0.13-0.73) | 0.43 (0.18-1.02) | 0.48 (0.20-1.16) |
| Sufficiently Active | 26/948 | 0.23 (0.14-0.40) | 0.38 (0.21-0.66) | 0.44 (0.24-0.79) | 15/613 | 0.32 (0.18-0.58) | 0.40 (0.22-0.74) | 0.47 (0.25-0.87) |
| *P* trend |  | <0.001 | 0.001 | 0.005 |  | 0.001 | 0.007 | 0.024 |

PA=physical activity; MVPA = moderate-vigorous intensity physical activity; HR=hazard ratio; CVD=cardiovascular disease.

Model 1: adjusted for age and sex; Model 2: also adjusted for COPD severity, self-reported longstanding illness and body mass index; Model 3: also adjusted for smoking status, education level and alcohol consumption.

^1^Participants who died within the first 12 months of follow-up were excluded from this analysis (n=49).

^2^Participants who reported no physical activity during the week; participants who reported no moderate-vigorous intensity physical activity during the week.

^3^Participants who reported <3.75 MET-hours/week of physical activity; participants who reported <75 minutes/week of moderate-intensity physical activity, or <32.5 minutes/week of vigorous-intensity physical activity, or an equivalent combination of moderate-vigorous intensity physical activity.

^4^Participants who reported between 3.75 and <7.5 MET-hours/week of physical activity; participants who reported between 75 and <150 minutes/week of moderate-intensity physical activity, or between 32.5 and <75 minutes/week of vigorous-intensity physical activity, or an equivalent combination of moderate-vigorous intensity physical activity.

^4^Participants who adhered to the current recommendation of ≥7.5 MET-hours/week of physical activity; participants who adhered to the current recommendation of ≥150 minutes/week of moderate-intensity physical activity, or ≥75 minutes/week of vigorous-intensity physical activity, or an equivalent combination of moderate-vigorous intensity physical activity.

**Table S2.** Sensitivity analysis of the associations of total physical activity and moderate-vigorous intensity physical activity with mortality risk in participants with COPD with a smoking history but no coexisting asthma or recent respiratory infection (n=1190)

|  | Total PA | | | | MVPA | | | |
| --- | --- | --- | --- | --- | --- | --- | --- | --- |
|  | **Cases/No.** | **Model 1**  **HR (95% CI)** | **Model 2**  **HR (95% CI)** | **Model 3**  **HR (95% CI)** | **Cases/No.** | **Model 1**  **HR (95% CI)** | **Model 2**  **HR (95% CI)** | **Model 3**  **HR (95% CI)** |
| All-cause mortality  (342 deaths) | | |  |  |  |  |  |  |
| Inactive ^1^ | 86/250 | 1.00 | 1.00 | 1.00 | 161/463 | 1.00 | 1.00 | 1.00 |
| Insufficiently Active (Low) ^2^ | 71/191 | 0.91 (0.66-1.24) | 0.97 (0.70-1.33) | 1.06 (0.77-1.46) | 78/240 | 0.73 (0.56-0.96) | 0.80 (0.60-1.05) | 0.84 (0.64-1.12) |
| Insufficiently Active (High)^3^ | 40/126 | 0.65 (0.44-0.94) | 0.71 (0.49-1.05) | 0.88 (0.59-1.31) | 32/125 | 0.55 (0.38-0.81) | 0.63 (0.43-0.92) | 0.70 (0.47-1.03) |
| Sufficiently Active^4^ | 145/623 | 0.52 (0.40-0.68) | 0.60 (0.45-0.79) | 0.70 (0.52-0.93) | 71/362 | 0.62 (0.47-0.82) | 0.71 (0.53-0.95) | 0.76 (0.57-1.02) |
| *P* trend |  | <0.001 | <0.001 | 0.002 |  | 0.005 | 0.054 | 0.124 |
| CVD mortality (110 deaths) |  |  |  |  |  |  |  |  |
| Inactive | 38/250 | 1.00 | 1.00 | 1.00 | 57/463 | 1.00 | 1.00 | 1.00 |
| Insufficiently Active (Low) | 22/191 | 0.63 (0.38-1.07) | 0.71 (0.42-1.21) | 0.76 (0.44-1.31) | 24/240 | 0.65 (0.40-1.04) | 0.78 (0.48-1.28) | 0.84 (0.51-1.37) |
| Insufficiently Active (High) | 7/126 | 0.26 (0.12-0.58) | 0.34 (0.15-0.78) | 0.40 (0.17-0.93) | 9/125 | 0.45 (0.22-0.91) | 0.65 (0.32-1.33) | 0.73 (0.35-1.52) |
| Sufficiently Active | 43/623 | 0.35 (0.23-0.55) | 0.50 (0.32-0.80) | 0.57 (0.35-0.92) | 20/362 | 0.49 (0.30-0.83) | 0.74 (0.43-1.27) | 0.80 (0.47-1.39) |
| *P* trend |  | <0.001 | 0.006 | 0.024 |  | 0.026 | 0.394 | 0.545 |
| Respiratory mortality  (52 deaths) | |  |  |  |  |  |  |  |
| Inactive | 15/250 | 1.00 | 1.00 | 1.00 | 29/463 | 1.00 | 1.00 | 1.00 |
| Insufficiently Active (Low) | 11/191 | 0.81 (0.37-1.77) | 1.05 (0.46-2.38) | 1.13 (0.50-2.56) | 12/240 | 0.56 (0.29-1.11) | 0.55 (0.27-1.14) | 0.64 (0.31-1.33) |
| Insufficiently Active (High) | 9/126 | 0.80 (0.35-1.84) | 0.81 (0.34-1.94) | 1.04 (0.41-2.62) | 2/125 | 0.20 (0.05-0.82) | 0.22 (0.05-0.92) | 0.24 (0.06-1.05) |
| Sufficiently Active | 17/623 | 0.37 (0.18-0.76) | 0.37 (0.18-0.78) | 0.43 (0.20-0.91) | 9/362 | 0.48 (0.22-1.01) | 0.41 (0.19-0.90) | 0.44 (0.20-0.99) |
| *P* trend |  | 0.005 | 0.003 | 0.012 |  | 0.113 | 0.063 | 0.078 |

PA=physical activity; MVPA = moderate-vigorous intensity physical activity; HR=hazard ratio; CVD=cardiovascular disease.

Model 1: adjusted for age and sex; Model 2: also adjusted for COPD severity, history of cardiovascular disease, history of cancer, history of diabetes, self-reported longstanding illness and body mass index; Model 3: also adjusted for smoking status, education level and alcohol consumption.

^1^Participants who reported no physical activity during the week; participants who reported no moderate-vigorous intensity physical activity during the week.

^2^Participants who reported <3.75 MET-hours/week of physical activity; participants who reported <75 minutes/week of moderate-intensity physical activity, or <32.5 minutes/week of vigorous-intensity physical activity, or an equivalent combination of moderate-vigorous intensity physical activity.

^3^Participants who reported between 3.75 and <7.5 MET-hours/week of physical activity; participants who reported between 75 and <150 minutes/week of moderate-intensity physical activity, or between 32.5 and <75 minutes/week of vigorous-intensity physical activity, or an equivalent combination of moderate-vigorous intensity physical activity.

^4^Participants who adhered to the current recommendation of ≥7.5 MET-hours/week of physical activity; participants who adhered to the current recommendation of ≥150 minutes/week of moderate-intensity physical activity, or ≥75 minutes/week of vigorous-intensity physical activity, or an equivalent combination of moderate-vigorous intensity physical activity.

**Table S3.** Sensitivity analysis of the associations of type-specific physical activity with mortality risk in participants with COPD without existing cardiovascular disease, cancer and diabetes at baseline^1^ (n=1635)

|  | **Walking** | | | | **Domestic PA** | | | | **Sport/exercise** | | | |
| --- | --- | --- | --- | --- | --- | --- | --- | --- | --- | --- | --- | --- |
|  | **Cases/No.** | **Model 1**  **HR (95% CI)** | **Model 2**  **HR (95% CI)** | **Model 3**  **HR (95% CI)** | **Cases/No.** | **Model 1**  **HR (95% CI)** | **Model 2**  **HR (95% CI)** | **Model 3**  **HR (95% CI)** | **Cases/No.** | **Model 1**  **HR (95% CI)** | **Model 2**  **HR (95% CI)** | **Model 3**  **HR (95% CI)** |
| **All-cause mortality**  **(391 deaths)** | | |  |  |  |  |  |  |  |  |  |  |
| No PA^2^ | 156/487 | 1.00 | 1.00 | 1.00 | 198/699 | 1.00 | 1.00 | 1.00 | 321/1139 | 1.00 | 1.00 | 1.00 |
| Low PA^3^ | 137/559 | 0.72 (0.57-0.90) | 0.79 (0.63-1.00) | 0.89 (0.70-1.13) | 97/421 | 0.69 (0.54-0.88) | 0.72 (0.56-0.92) | 0.76 (0.60-0.98) | 37/235 | 0.68 (0.48-0.96) | 0.74 (0.53-1.04) | 0.76 (0.54-1.08) |
| High PA^4^ | 98/589 | 0.49 (0.38-0.63) | 0.55 (0.42-0.71) | 0.64 (0.49-0.84) | 96/515 | 0.72 (0.57-0.93) | 0.76 (0.59-0.97) | 0.81 (0.63-1.05) | 33/261 | 0.49 (0.34-0.70) | 0.54 (0.37-0.77) | 0.58 (0.40-0.84) |
| *P* trend |  | <0.001 | <0.001 | 0.001 |  | 0.037 | 0.082 | 0.217 |  | <0.001 | 0.002 | 0.008 |
| **CVD mortality**  **(101 deaths)** | | |  |  |  |  |  |  |  |  |  |  |
| No PA | 44/487 | 1.00 | 1.00 | 1.00 | 55/699 | 1.00 | 1.00 | 1.00 | 89/1139 | 1.00 | 1.00 | 1.00 |
| Low PA | 33/559 | 0.62 (0.40-0.98) | 0.65 (0.41-1.02) | 0.74 (0.46-1.18) | 20/421 | 0.51 (0.31-0.85) | 0.51 (0.31-0.86) | 0.54 (0.32-0.91) | 6/235 | 0.41 (0.18-0.94) | 0.42 (0.19-0.97) | 0.43 (0.19-0.98) |
| High PA | 24/589 | 0.44 (0.27-0.72) | 0.48 (0.29-0.80) | 0.56 (0.33-0.95) | 26/515 | 0.73 (0.46-1.16) | 0.71 (0.44-1.15) | 0.78 (0.49-1.27) | 6/261 | 0.33 (0.14-0.76) | 0.37 (0.16-0.85) | 0.38 (0.17-0.89) |
| *P* trend |  | 0.002 | 0.008 | 0.039 |  | 0.421 | 0.373 | 0.620 |  | 0.030 | 0.054 | 0.066 |
| **Respiratory mortality**  **(87 deaths)** | |  |  |  |  |  |  |  |  |  |  |  |
| No PA | 38/487 | 1.00 | 1.00 | 1.00 | 47/699 | 1.00 | 1.00 | 1.00 | 77/1139 | 1.00 | 1.00 | 1.00 |
| Low PA | 34/559 | 0.72 (0.45-1.15) | 0.97 (0.60-1.58) | 1.13 (0.68-1.88) | 25/421 | 0.71 (0.44-1.16) | 0.70 (0.42-1.16) | 0.79 (0.48-1.32) | 3/235 | 0.23 (0.07-0.73) | 0.30 (0.10-0.97) | 0.34 (0.11-1.09) |
| High PA | 15/589 | 0.30 (0.16-0.54) | 0.39 (0.21-0.73) | 0.48 (0.25-0.93) | 15/515 | 0.47 (0.26-0.84) | 0.53 (0.29-0.97) | 0.60 (0.33-1.11) | 7/261 | 0.42 (0.20-0.92) | 0.53 (0.24-1.15) | 0.64 (0.29-1.42) |
| *P* trend |  | <0.001 | 0.002 | 0.016 |  | 0.017 | 0.057 | 0.122 |  | 0.166 | 0.322 | 0.581 |

PA=physical activity; HR=hazard ratio; CVD=cardiovascular disease.

Model 1: adjusted for age and sex; Model 2: also adjusted for COPD severity, self-reported longstanding illness and body mass index; Model 3: also adjusted for smoking status, education level, alcohol consumption and the two other type-specific PA variables.

^1^Participants who died within the first 12 months of follow-up were excluded from this analysis (n=49).

^2^“No PA” was defined as no self-reported walking during the week, no self-reported domestic physical activity during the week, and no self-reported sport/exercise during the week.

^3^“Low PA” was defined as <5.25 MET-hours/week of walking, <5.70 MET-hours/week of domestic physical activity, and <8.00 MET-hours/week of sport/exercise.

^4^“High PA” was defined as ≥5.25 MET-hours/week of walking, ≥5.70 MET-hours/week of domestic physical activity, and ≥8.00 MET-hours/week of sport/exercise.

**Table S4.** Sensitivity analysis of the associations of type-specific physical activity with mortality risk in participants with COPD with a smoking history but no coexisting asthma or recent respiratory infection (n=1190)

|  | **Walking** | | | | **Domestic PA** | | | | **Sport/exercise** | | | |
| --- | --- | --- | --- | --- | --- | --- | --- | --- | --- | --- | --- | --- |
|  | **Cases/No.** | **Model 1**  **HR (95% CI)** | **Model 2**  **HR (95% CI)** | **Model 3**  **HR (95% CI)** | **Cases/No.** | **Model 1**  **HR (95% CI)** | **Model 2**  **HR (95% CI)** | **Model 3**  **HR (95% CI)** | **Cases/No.** | **Model 1**  **HR (95% CI)** | **Model 2**  **HR (95% CI)** | **Model 3**  **HR (95% CI)** |
| **All-cause mortality**  **(342 deaths)** | | | |  |  |  |  |  |  |  |  |  |
| No PA^1^ | 133/396 | 1.00 | 1.00 | 1.00 | 183/586 | 1.00 | 1.00 | 1.00 | 283/870 | 1.00 | 1.00 | 1.00 |
| Low PA^2^ | 105/389 | 0.76 (0.59-0.97) | 0.83 (0.64-1.07) | 0.93 (0.71-1.21) | 61/291 | 0.72 (0.55-0.93) | 0.79 (0.60-1.03) | 0.91 (0.69-1.21) | 15/152 | 0.62 (0.42-0.91) | 0.63 (0.43-0.93) | 0.70 (0.47-1.04) |
| High PA^3^ | 72/405 | 0.54 (0.41-0.70) | 0.58 (0.44-0.77) | 0.69 (0.51-0.92) | 64/313 | 0.84 (0.64-1.10) | 0.93 (0.71-1.23) | 1.06 (0.80-1.42) | 36/168 | 0.56 (0.38-0.81) | 0.62 (0.42-0.91) | 0.71 (0.48-1.05) |
| *P* trend |  | <0.001 | <0.001 | 0.009 |  | 0.408 | 0.875 | 0.569 |  | 0.010 | 0.045 | 0.169 |
| **CVD mortality (110 deaths)** |  |  |  |  |  |  |  |  |  |  |  |  |
| No PA | 133/396 | 1.00 | 1.00 | 1.00 | 183/586 | 1.00 | 1.00 | 1.00 | 283/870 | 1.00 | 1.00 | 1.00 |
| Low PA | 105/389 | 0.54 (0.35-0.83) | 0.66 (0.42-1.03) | 0.70 (0.44-1.12) | 61/291 | 0.55 (0.34-0.90) | 0.69 (0.42-1.14) | 0.84 (0.50-1.42) | 15/152 | 0.25 (0.09-0.67) | 0.27 (0.10-0.72) | 0.31 (0.11-0.84) |
| High PA | 72/405 | 0.38 (0.24-0.62) | 0.49 (0.30-0.80) | 0.55 (0.33-0.93) | 64/313 | 0.76 (0.48-1.22) | 1.05 (0.64-1.73) | 1.31 (0.78-2.19) | 36/168 | 0.43 (0.21-0.89) | 0.55 (0.27-1.15) | 0.65 (0.31-1.37) |
| *P* trend |  | <0.001 | 0.007 | 0.037 |  | 0.517 | 0.602 | 0.230 |  | 0.134 | 0.393 | 0.617 |
| **Respiratory mortality**  **(52 deaths)** | |  |  |  |  |  |  |  |  |  |  |  |
| No PA | 133/396 | 1.00 | 1.00 | 1.00 | 183/586 | 1.00 | 1.00 | 1.00 | 283/870 | 1.00 | 1.00 | 1.00 |
| Low PA | 105/389 | 0.92 (0.49-1.71) | 0.95 (0.49-1.87) | 1.40 (0.68-2.87) | 61/291 | 0.69 (0.37-1.28) | 0.64 (0.33-1.24) | 0.87 (0.41-1.81) | 15/152 | 0.61 (0.22-1.71) | 0.61 (0.21-1.72) | 0.74 (0.25-2.16) |
| High PA | 72/405 | 0.51 (0.25-1.07) | 0.50 (0.23-1.09) | 0.79 (0.34-1.82) | 64/313 | 0.43 (0.18-1.03) | 0.38 (0.15-0.93) | 0.38 (0.15-0.98) | 36/168 | 0.51 (0.18-1.44) | 0.58 (0.21-1.63) | 0.73 (0.24-2.17) |
| *P* trend |  | 0.062 | 0.063 | 0.044 |  | 0.077 | 0.048 | 0.045 |  | 0.279 | 0.398 | 0.638 |

PA=physical activity; HR=hazard ratio; CVD=cardiovascular disease.

Model 1: adjusted for age and sex; Model 2: also adjusted for COPD severity, history of CVD, history of cancer, history of diabetes, self-reported longstanding illness and body mass index; Model 3: also adjusted for smoking status, education level, alcohol consumption and the two other type-specific PA variables.

^1^“No PA” was defined as no self-reported walking during the week, no self-reported domestic physical activity during the week, and no self-reported sport/exercise during the week.

^2^“Low PA” was defined as <5.25 MET-hours/week of walking, <5.70 MET-hours/week of domestic physical activity, and <8.00 MET-hours/week of sport/exercise.

^3^“High PA” was defined as ≥5.25 MET-hours/week of walking, ≥5.70 MET-hours/week of domestic physical activity, and ≥8.00 MET-hours/week of sport/exercise.
